# Data Collection in IoT Using UAV Based on Multi-Objective Spotted Hyena Optimizer

**DOI:** 10.3390/s22228896

**Published:** 2022-11-17

**Authors:** Hamza Mohammed Ridha Al-Khafaji

**Affiliations:** Biomedical Engineering Department, Al-Mustaqbal University College, Hillah 51001, Babil, Iraq; hamza.alkhafaji@uomus.edu.iq

**Keywords:** internet of things, unmanned aerial vehicle, multi-objective optimization, spotted hyena optimizer

## Abstract

Today, the use of information and communication technology is very important in making the internet of things (IoT) elements distributable around the earth. With the development of IoT topics, today unmanned aerial vehicles (UAV) are utilized as a platform for gathering data from various IoT devices located worldwide. Determining the number and optimal locations of drones can minimize energy consumption in this data-collection system in the IoT. Using a promising multi-objective optimization algorithm (MOA) can achieve this goal. In this research, a bio-inspired MOA, termed the multi-objective spotted hyena optimizer (MOSHO), is employed on the data-collection platform for a group of IoT devices in a geographical area. The results of this method have been compared with other evolutionary MOAs. The analysis of the results shows that the MOSHO has a noteworthy consequence on the process of optimal energy consumption in this system, in addition to a high convergence associated with better diversity and robustness. The results of this research can be used to identify the optimization parameters in this system.

## 1. Introduction

Today, the internet of things (IoT) has taken over the world of information and communication technology, and has affected all aspects of human life with smart solutions [1]. This technology is presented as creating a more efficient, beautiful, and comfortable future. To better understand the concept of the IoT, this technology refers to many objects and devices around us that can be recognized, located, addressed, and controlled through a network [2]. In simple terms, with this technology, sensors and devices can interact with each other and with their users within a network.

Currently, most organizations and businesses are making extensive use of this technology [3]. Among these industries are the aviation and airport industry, banking and security, construction, urban development, medicine, etc. [4]. By benefiting from the IoT’s advantages, they gain a better understanding of their audience and can provide better and more appropriate services [5]. Furthermore, the decision-making processes in various issues is improved by using the capabilities of the IoT.

The IoT is a collection of all of the physical things that are connected to the Internet in some way [6]. This collection includes everything from mobile phones and vehicles to buildings, sensors, and all other items equipped with electronics that can help users to do different things [7]. Lately, drones have also joined the IoT collection with the help of secure connections and reliable wireless Internet. These drones can be controlled directly, and their missions can even be changed in the middle of a flight [8].

As the level of drone technology improves, the ability of drones to interact with their environments and respond to them also increases. The existence of drones that will be able to be sent to different places and carry flexible loads also improves. The drone can reprogram, measure, and provide analytical data about anything at any time [9]. The technology of these small aircraft is constantly advancing, and more and more business owners are finding ways to use drones in their daily activities.

In areas with weak terrestrial wireless networks, IoT devices with limited capacities cannot communicate the data remotely to a base station [10]. Consequently, efficient data collection from IoT devices is a challenging issue [11]. Today, drones are widely used due to their mobility, autonomy, communication, processing power, relatively low cost (because they eliminate all of the risks related to human operators), and because they cost much less than manned aircraft [10]. Furthermore, the use of unmanned aerial vehicles (UAVs) as emerging platforms for gathering information has been widely considered.

Compared with terrestrial data-collection platforms, UAVs’ data-collection platforms provide various supremacies: (1) UAVs are able to contribute more obediently to the goals of campaigns because of their mobility and agility; (2) UAVs can create remote-viewing connections to benefit the aims of campaigns; (3) UAVs offer emergency services support for the aims of campaigns during unanticipated incidents. Therefore, the application of drones is suitable for delivering a satisfying method for gathering data from IoT devices.

The optimization of UAV deployment has already been studied. In 2018, Chen et al. [12] proposed a 3D settlement of UAV base stations using an improved genetic algorithm to provide the maximum number of users with diverse quality-of-service needs [13]. For maximizing the quantity of users that is covered, the 3D placement problem in the study was represented as a placement problem involving numerous concentric rings. First, the features of the problem were emphasized [14]. Then, after performing some mathematical operations to decouple the UAV base station implementation problem in horizontal and vertical proportions without sacrificing optimality, the mixed-integer second-order cone problem was formulated, and an enhanced multi-population genetic algorithm (MPGA) was suggested for the assignment problem in the horizontal dimensions. The performance of the modified MPGA in comparison with the standard genetic algorithm (SGA) was demonstrated through computational models. However, IoT technology, on the other hand, would have enabled smarter system control and improved personal safety if the strategy had been employed.

Özdağ et al. [15] presented a new optimal methodology for the 3D assignment of multiple UAV base stations in wireless networks. The optimization method was based on a combination of a maximum drone-deployment algorithm (MDDA) and electromagnetism-like (EML) algorithms. For assessing the efficiency of the created MDDA–EML, pure-EML, using the fundamental EML algorithm, was also built. Based on the experimental results, MDDA–EML, which covered the most customers in the field and achieved the highest handling rates in comparison with pure-EML, was able to effectively organize many 3D drone base stations at close to the optimal height. This high-level conflict approach’s main flaw was that it went into great depth on how certain classes, procedures, and items were implemented. Additionally, the implementation of business logic was not related to the object-oriented paradigm; therefore, the programmer must write a ton of code to compensate for this.

Na et al. [16] presented a multicarrier wireless-powered communication model with UAV assistance for IoT applications. In particular, the UAV acted as an airborne base station, transmitting orthogonal frequency-division multiplexing (OFDM) signals to the IoT nodes, which in turn decoded data and collected the signals’ energy. Afterwards, the IoT nodes used the collected energy to communicate data to the UAV. On the basis of OFDM, a suggested combined UAV-trajectory-optimization approach and resource allocation was put forth. By concurrently optimizing the UAVs’ trajectory, power, sub-slot allocation, and subcarrier subject to the attainable total rate of all of the IoT nodes in the downlink, the goal was to maximize the least-attainable rate in the uplink among all of the IoT nodes. An alternate-iteration approach was suggested to solve the problem due to the complicated nonconvex optimization nature. The proposed technique can optimize the UAV trajectory and regulate node movement with regards to the simulation results. The suggested system not only greatly improves the lowest attainable rate when compared with standard resource-allocation techniques, but it also functions effectively for two flying modes. Nonetheless, this technique has some disadvantages. Systems using drone swarms usually do not consider the low-cost solution.

Masroor et al. [17] introduced resource management in UAV-aided wireless networks. From an optimization viewpoint, this study offered a thorough analysis of resource allocation in UAV-assisted wireless networks. To emphasize the relevance of the UAV-assisted wireless networks, the classification of UAVs, their advantages, and their applications were explained. The management of resources was then covered in depth, with metrics such as UAV placement, backhaul, trajectory, spectrum, path planning, data offloading, and charging included. There was also a discussion of various optimization types, limitations, and solutions designed to enable UAV-assisted wireless networks. In order to solve the issues preventing the successful implementation of UAV-assisted wireless networks, the study concluded by offering future research options. Despite their method’s advantages, the method can be improved by adding more aims, such as placement problems, multi-UAV-assisted wireless networks, and multi-objective data collection.

To use the drone efficiently, the deployment of the drone should be optimized. If organized correctly, a drone must be able to deliver a dependable and low-cost solution for IoT data. Different multi-objective optimization techniques have been paid attention, such as the well-developed epsilon constraint approach [18], the weighted sum method [19], and the interactive fuzzy-programming scheme [20]. Moreover, the concept of synchro-modal and intermodal are among the important solutions in routing optimization [21,22]. In order to explore the optimum solution, a bio-inspired multi-objective optimization algorithm (MOA) is utilized on the platform of data collection for a group of IoT devices across the earth. The next sections of the paper are organized as follows: Section 2 presents the materials and methods for data collection in the IoT, which is based on multi-objective spotted hyena optimizer (MOSHO). Section 3 describes the theoretical modelling of the study. Section 4 discusses the simulation results of using the method on the case study, and finally, the paper is concluded in Section 5.

## 2. Materials and Methods

Nature-inspired algorithms have been presented as an effective tool for exploring convergent and divergent Pareto optimal fronts in multi-objective optimization problems, which generally include two or three conflicting objectives. Multi-objective optimization problems are related to solving M conflicting objectives at the same time in problems where the value of M is equal to two or three. In general, a multi-objective optimization problem has the following relationship which is expressed as follows:

(1) $$F\left(x\right)=\left[f_{1}\left(x\right),\;f_{2}\left(x\right),\ldots ,f_{n}\left(x\right)\right]$$

where x∈K and $K\in {\mathbb{R}}^{n}$ are the decision space and $f:K\to \Omega \in {\mathbb{R}}^{n}$ is the search space, and it is assumed that f is a minimization problem. Most multi-objective nature-inspired algorithms are focused on maintaining diversity and increasing convergence. In this study, a MO form of a spotted hyena optimizer was designed and utilized to achieve the research aim.

### 2.1. Spotted Hyena Optimizer

In this section, the fundamental notions of a spotted hyena optimizer (SHO) are introduced before moving on to a brief overview of the MO variant of a spotted hyena optimizer. The fundamental idea of SHO is initially presented, before the multi-objective variant of SHO is suggested [23,24]. The primary sources of inspiration for this algorithm were the social interactions and hunting habits of spotted hyenas. The SHO imitates the dependable spotted hyenas’ coherent groups. Searching, surrounding, hunting, and attacking are the four fundamental components of SHO. The SHO algorithm uses a group of trusted people to direct the hunting behavior toward the most efficient search agent and store the finest optimum solutions. The following formula is utilized to model the spotted hyena’s circling pattern:

(2) $$D_{h}=\left|\vec{B}\cdot {\vec{X}}_{P}\left(x\right)-\vec{X}\left(x\right)\right|$$

(3) $$\vec{X}\left(x+1\right)={\vec{X}}_{P}\left(x\right)-\vec{E}\cdot {\vec{D}}_{h}$$

where $\vec{D_{h}}$ stands for the separation between both the target and the spotted hyena. The iteration is indicated by x. The location vectors of the spotted hyena and its target are represented by $\vec{X_{p}}$ and X, respectively, and || is the absolute value. The co-efficient vectors are $\vec{B}$ and $\vec{E}$. The formulas used to evaluate $\vec{B}$ and $\vec{E}$ are as follows:

(4) $$\vec{B}=2\cdot r{\vec{d}}_{1}$$

(5) $$\vec{E}=2\vec{h}\cdot r{\vec{d}}_{2}-\vec{h}$$

(6) $$\vec{h}=5-(Iteration\times \frac{5}{Max_{iteration}})$$

where iteration is between one to maximum iteration.

In this case, $\vec{h}$ showed gradual decreases throughout the duration of iterations from 5 to 0. The stochastic arrays $\vec{rd_{1}}$ and $\vec{rd_{2}}$ were those that fell between [0, 1]. By changing the values of vectors $\vec{B}$ and $\vec{E}$, other locations could be achieved relative to the present location. This algorithm pushed other search agents to improve their locations while saving the optimal answer so far.

The following equations were created to replicate the hunting behavior of spotted hyenas and identify viable random search areas:

(7) $${\vec{D}}_{h}=\left|\vec{B}\cdot {\vec{X}}_{h}-{\vec{X}}_{k}\right|$$

(8) $${\vec{X}}_{k}={\vec{X}}_{h}-\vec{E}\cdot {\vec{D}}_{h}$$

(9) $${\vec{C}}_{h}={\vec{X}}_{k}+{\vec{X}}_{k+1}+\ldots +{\vec{X}}_{k+N}$$

where N is the number of iterations, which may be computed as:

(10) $$N=count_{nos}({\vec{X}}_{h}+{\vec{X}}_{h+1}+{\vec{X}}_{h+2},\ldots \ldots ,{\vec{X}}_{h+\vec{M}})$$

(11) $${\vec{X}}_{\left(x+1\right)=}\frac{{\vec{C}}_{h}}{N}$$

where nos is the number of possible solutions in the given solution space which are very similar to the best optimum solution, ${\vec{C}}_{h}$ denotes a collection or cluster of optimal solutions, and $\vec{M}$ denotes a random vector in the range between 0.5 and 1. ${\vec{X}}_{\left(x+1\right)}$ changes the locations of additional search agents based on the location of the successful completion of this work and records the N  most optimum answers.

**Figure 1:**
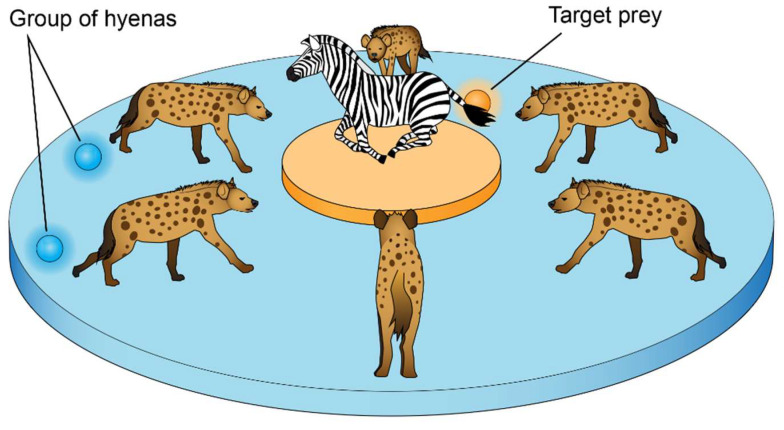
The process of attacking to the prey by the hyena hunters.

The search candidates are required to retreat from the target, as illustrated in Figure 1 by the exploration criteria that were met through vector $\vec{E}$ with random values larger than 1 or less than 1. $\vec{B}$, another component of SHO, is in charge of exploration [25]. It supplies the weight of the prey and has random values between [0, 5]. When |$\vec{E}$| < 1, the SHO method can be used for attacking the prey, as shown in Figure 1, by meeting the prey’s estimated location.

The method starts by producing a collection of random answers as the population in order to execute the optimization. The search agents group toward the best search agent throughout the optimization.

Vectors $\vec{h}\;$ and $\vec{E}$, however, continuously decline during the course of repetitions. When the terminating requirement is met, the placements of candidate solutions that form a cluster are finally regarded as the best optimum solutions. The SHO technique is capable of effectively resolving optimization issues. SHO’s enormous exploration capacity yields superior outcomes than the current metaheuristics. As a result, SHO’s answers do not become locked-in optimal answers. This is what spurred the author to utilize the MOSHO.

### 2.2. Multi-Objective Spotted Hyena Optimizer (MOSHO)

Multi-objective optimization defines a field of multiple-criteria decision making, which includes more than one objective function to be optimized simultaneously. To construct a MO-based model of SHO, two components are included. These features are very comparable to those of MOSHO. The strongest non-dominated Pareto optimum answer is stored in the first component, an archive [26]. The second element uses a clustering method of selecting and choosing close solutions from the collection that match the location of the target. The sub-section before this one discusses these parts. Algorithm 1 indicates the description of MOSHO [27].
**Algorithm 1**. MOSHO1.Initialization of $\vec{h}\;$, $\vec{B}$, $\vec{E}$, and N2.Evaluate the objective values of each search agent3.Obtain all of the non-dominated solutions4.nitialize ${\vec{X}}_{h}$ from archive and ${\vec{C}}_{h}$ according to ${\vec{X}}_{h}$5.While ($x<Max_{iteration}$)6.Update the position by Equations (7)–(11)7.Update $\vec{h}\;$, $\vec{B}$, $\vec{E}$, N, and ${\vec{C}}_{h}$ according to ${\vec{X}}_{h}$8.x=x+19.End while10.Return the solution

### 2.3. Archive

All so-far-discovered non-dominated Pareto optimum solutions have an archive as a storage component. In the case of concave, convex, and unconnected Pareto fronts, this archive component has the capacity to disperse uniformly. There are two primary components, such as array and storage manager.

### 2.4. The Archive Controller

This operator’s primary duty is to determine whether or not the answer should be archived. The following are several crucial details about the archive updating procedure.

Whenever the archive is managed by at minimum one archive member, the answer should not be permitted to enter.

- After excluding the dominated solution(s) from the archive, a new answer that governs one or more associates of the archive will be permitted to arrive at the archive.
- If neither the new answer nor the archive answers overwhelm one another, the archive should receive a new member.
- Whenever the archive is complete, the grid approach should be used to exclude one of the answer sections which is the maximum packed and to add a new answer to increase the diversity, according to the Pareto optimum front.

### 2.5. The Grid

The scattered Pareto fronts are created utilizing an adaptive grid approach. The objective function space is separated into several areas. If a population inserts a person outside of the grid’s existing boundaries, the grid must be recalculated, and each person relocated. The adaptive grid is a hypercube-based environment that is employed to distribute information uniformly.

### 2.6. Group-Selection Mechanism

The difficult challenge in a multi-objective search space is to evaluate the answers with archive members. A technique for group answers was created to address this problem. The decision was made using the probability-based roulette-wheel technique, which is described as:

(12) $$H_{k}=\frac{f}{S_{k}}$$

where S is the amount of Pareto optimum answers that have been found thus far in the kth segment and f is a constant number that should be bigger than one [27]. A well-known selection mechanism of the proportional kind is the roulette-wheel approach. Every person in the population has an optimal solution that resembles the proportional region on a roulette wheel. The MOSHO (see Algorithm 1) incorporates the characteristics of the SHO algorithm. The primary distinction between SHO and MOSHO is that the former searches inside a set of archive members while the latter saves the collection of best-case scenarios.

The archive is where NSGA-2 and MOSHO diverge most. The goal of using archives is to lessen the likelihood that non-dominated answers would deteriorate. Comparatively speaking with other archive-based techniques, MOSHO offers the best options. Standard selection operators used by archive-based algorithms include crossover, mutation, etc. These controllers lean the search in favor of archive members. Parameters are swapped inside of a solution in the search space in MOSHO. The swap procedure improves MOSHO’s capacity for exploration. As opposed to that, it lessens the converging feature. In order to find a minimum of non-dominated solutions in the available exploration area, MOSHO employs a cluster decision model to solve this issue.

## 3. Conceptual Model of the Research

### 3.1. Model Conception

In this research, the positions and the quantity of stopping arguments are instantaneously optimized for UAV placement. Due to the fact that the number of stopping points is unknown and not specified in advance, and must therefore be varied throughout the optimization process, the issue creates a great challenge for the traditional gradient, as no clear description of the slope vector is present. Evolutionary algorithms have the ability to deal with this problem.

In this research, the IoT’ information-collection system has been studied with the help of a drone. Here, the positions and the quantity of stopping arguments are simultaneously optimized for drone placement, which leads to the provision of better services for the IoT devices and achieves more energy-efficient data collection. Due to the fact that the length of people is fixed, the MOSHO algorithm for optimizing UAV deployment does not need to use operators such as intersection and mutation that exist in the genetic algorithm. Based on [28], it is verified that the UAV deployment problem is an NP-hard problem. Each person’s length is also decreased by two. Thus, in a two-dimensional search space, the population is seeking the ideal distribution of halting locations.

**Figure 2:**
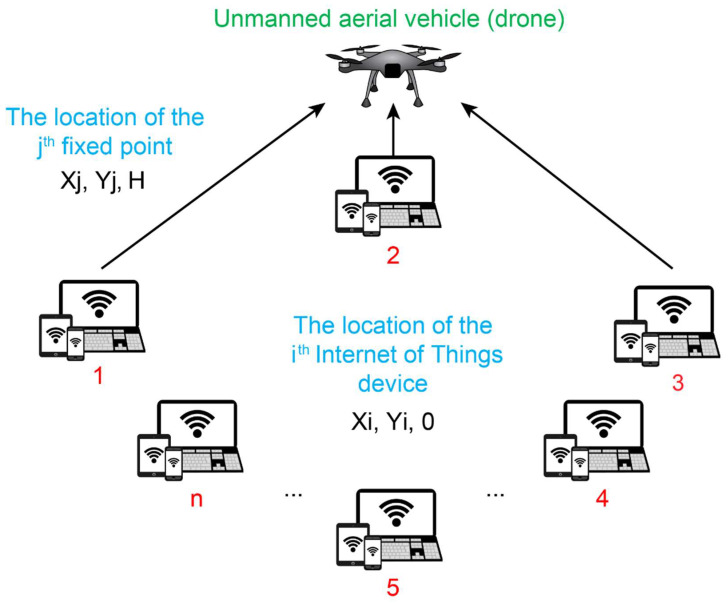
Conceptual model of the research.

A new approach is being developed to modify the population size (i.e., the number of stopping points). In addition, depending on the improving efficiency, the number of breakpoints might be raised, dropped, or left constant in each update. This results in the omission of a crucial parameter, namely the size of the population. Moreover, the MOSHO algorithm acts as a search engine for the position-optimization of the stop points. Figure 2 shows the conceptual model of the research.

According to Figure 2, the conceptual model is an IoT information-collection system with the help of a drone, which includes a rotary-wing UAV and a collection of n ground-based IoT devices with $N=\left[1,2,\ldots ,n\right]$ being shown. These IoT devices’ data are collected using a UAV as a framework for flight data collecting. The UAV can move the stations’ positions numerous times because of its flexibility and movement, which increases coverage while using less energy.

Here, it is assumed that the number of stopping points is k and that no prior information is available. The breakpoints can be set as $K=\left[1,2,\ldots ,k\right]$.

### 3.2. Fitness Function

The coordinates of the *i*th IoT devices are recognized and immobile ($X_{i}$, $Y_{i}$, 0) where $X_{i}$ and $Y_{i}$ are the values of the x and y coordinate vectors of the *i*th IoT device. Furthermore, it is assumed that the UAV is hovering at a static height H, and the position of the fixed point (j∈K) is shown by ($X_{j},\;Y_{j}\;,\;H$) where $X_{j}$ and $Y_{j}$ are the values of the x and y coordinate vectors corresponding to the *j*th fixed point. Therefore, the distance between the IoT’ arguments and the stopping point j is defined below:

(13) $$d_{ij}=\sqrt{{\left(X_{j}-X_{i}\right)}^{2}+{\left(Y_{j}-Y_{i}\right)}^{2}+H^{2}}\;\forall i\in N,\;j\in K$$

A binary variable is used to represent the connection between the drone and the IoT’ elements at the drone’s halting position. If the IoT device provides data to the drone at a fixed location, the value of this variable is equal to 1; else, it is equal to 0. Every IoT device always sends data to the closest stop-point to conserve energy during transmission. So:

(14) $$C_{1}:a_{ij}=\left\{\begin{array}{c} 1,\;\;\;if\;j=\arg\min d_{ij}\\ 0,\;\;\;otherwise \end{array}\right.$$

(15) $$C_{2}=\sum_{j=1}^{K}a_{ij}=1$$

where i∈N and j∈K.

Consequently, all IoT arguments select a single breakpoint to transfer their data to. Additionally, each stopping point’s UAV can acknowledge IoT devices to transfer signals instantaneously due to the system’s bandwidth restriction. Therefore:

(16) $$C_{3}=\sum_{i=1}^{m}a_{ij}\leq M$$

To ensure the service of all IoT devices, the following conditions must be met:

(17) $$C_{4}=\sum_{i=1}^{m}\sum_{j=1}^{K}a_{ij}=m$$

In the proposed model, the channel amplification between the drone at the *j*th stopping point and the IoT device is calculated using the following equation:

(18) $$h_{ij}=h_{0}d_{ij}^{-2}=\frac{h_{0}}{{\left(X_{j}-X_{i}\right)}^{2}+{\left(Y_{j}-Y_{i}\right)}^{2}+H^{2}}$$

Consequently, if the IoT device directs data to the drone at stop point j, the data rate is calculated by the following relation:

(19) $$r_{ij}=B\log_{2}\left(1+\frac{p_{i}h_{ij}}{\sigma^{2}}\right)=B\log_{2}\left(1+\frac{p_{i}h_{0}}{\sigma^{2}\left({\left(X_{j}-X_{i}\right)}^{2}+{\left(Y_{j}-Y_{i}\right)}^{2}+H^{2}\right)}\right)$$

where $p_{i}$ defines the transmission power from the *i*th IoT device to the drone. The $h_{0}$ indicates the amplification of the channel’s power in the reference distance $d_{0}=2m$. $\sigma^{2}$ stands for the power of Gaussian white noise and B is the bandwidth of the system. 

It is assumed that the *i*th IoT device delivers $D_{i}$ amount of the data sent to the drone. The time to send data from the *i*th IoT device to the drone at the *j*th stopping point is calculated as follows:

(20) $$T_{ij}=\frac{D_{i}}{r_{ij}},\forall i\in N,\;j\in K$$

Additionally, the energy consumption is calculated as follows:

(21) $$E_{ij}=p_{i}T_{ij}=\frac{p_{i}D_{i}}{r_{ij}},\;\forall i\in N,j\in K$$

Therefore, it is possible to obtain the energy consumption of all IoT devices:

(22) $$E_{\mathrm{IoT}}=\sum_{i=1}^{m}\sum_{j=1}^{K}a_{ij}E_{ij}$$

In fact, the drone will hover for a while at each stop point. Until the collection of all of the data is sent from the IoT devices to this stop point, it will not be transferred to another stop point.

Consequently, the drone floating time at the stopping point j is calculated as follows:

(23) $$T_{j}^{h}=\underset{i\in n}{max}\left[a_{ij}T_{ij}\right]$$

Furthermore, the floating energy consumption of the UAV at the *j*th the stopping point is calculated as follows:

(24) $$E_{j}^{h}=p^{h}T_{j}^{h}$$

where $p^{h}$ shows the drone’s floating power. 

Therefore, the drone’s total energy consumption can be calculated as follows:

(25) $$E_{\mathrm{UAV}}=\sum_{j=1}^{K}E_{j}^{h}$$

Here, the drone flight’s energy consumption of the UAV flight is ignored. The energy consumption of the system consists of the energy consumption of the drone and all IoT devices. Therefore, the issue can be related as follows:

(26) $$E_{\mathrm{UAV}}=\sum_{j=1}^{K}E_{j}^{h}$$

Here, the drone flight’s energy consumption is ignored. This term consists of the energy consumption of the drone and all IoT devices. Therefore, the issue (fitness function) can be related as follows:

(27) $$\underset{\left[X_{j},Y_{j}\right],k\;}{min}E_{\mathrm{UAV}}+\gamma E_{\mathrm{IoT}}$$

Subject to:
$C_{1}:a_{ij}\in \left[0,\;1\right],\;\forall i\in N$, j∈K$C_{2}:\sum_{j=1}^{n}a_{ij}=1$, ∀i∈N$C_{3}:\sum_{j=1}^{n}a_{ij}\leq M$, ∀i∈N$C_{4}:\sum_{i=1}^{m}\sum_{j=1}^{K}a_{ij}=n$$C_{5}:X_{min}\leq X_{j}\leq X_{max}$,∀j∈K$C_{6}:Y_{min}\leq Y_{j}\leq Y_{max}$,∀j∈K$C_{7}:k_{min}\leq k\leq k_{max}$

where γ≥0 describes the weight between the UAV’s energy consumption and all IoT devices. $X_{min}$ and $X_{max}$ represent, in turn, the inferior and the superior bounds of $X_{j}$. $Y_{min}$ and $Y_{max}$, represent, in turn, the inferior and the superior bounds of $Y_{j}$. $k_{min}$ and $k_{max}$ are, in turn, the inferior and the superior values of k. Hence, when the number of IoT devices working is no less than one IoT device at a stopping point, the $k_{min}$ and $k_{max}$ are, in turn, fit for $\left|\frac{n}{M}\right|$ and n.

Here, the aim is to optimize drone arrangement, as well as the positions and the number of drone stations, to achieve minimum system energy consumption in all constraints. Actually, the objective is to optimize $\left(X_{1},Y_{1},\ldots ,X_{k},Y_{k}\right)$ to achieve the minimum value of ($E_{\mathrm{UAV}}+\gamma E_{\mathrm{IoT}}$) while satisfying $C_{1}$ to $C_{7}$. The aim of this research is to optimize the position and the quantity of the station in the deployment of the drone jointly. Basically, the MOSHO algorithm obtains the optimization of the stop points’ quantity by adaptively updating the size of the population. Furthermore, the stopping places were optimized. The evolutionary MOA based on inverse modeling using Gaussian processing treats the entire population as a total deployment, and the position changes at most one breakpoint in each update. The new breakpoint location is created by inverse density estimation, which allows for the use of information from other breakpoint locations. As a result, global search capability is better than local search. The algorithm will be stopped when the iteration number has been reached.

## 4. Simulation Results

The approach to the solving of a problem is one of the most basic aspects of research, which is assigned to this section. According to the definition and mathematical model of the problem of this research, the solution method that is considered for it is the evolutionary multi-objective algorithm based on inverse modeling using the Gaussian process. Numerous heuristic methods have been created in the last three decades to handle multi-objective optimization issues. Nevertheless, it might be difficult for academics to accurately assess current algorithms and to employ specific algorithms to address issues in the absence of an up-to-date and extensive software platform.

In this study, PlatEMO, a system that is based on the MATLAB software for metaheuristic-based MO optimization, was employed to avoid this difficulty, because the source code for different methods has not been made publicly available. Together with numerous frequently utilized functions, it offers more than fifty multi-objective optimization techniques and more than one hundred multi-objective test tasks. This platform is completely open source, so users can build new algorithms based on it. The plateEMO source code can be accessed at [14]. This software is the basis of the exact solution for the proposed model of this research, and the goal is to validate the model. In the research findings, the proposed model will be validated with a precise method using this software. This research is developmental in terms of its purpose because it seeks to find a suitable scientific method to solve a problem.

In terms of the nature of the data, it is mixed (quantitative and qualitative), and in terms of the data collection method, it is also descriptive. The research method in this study is descriptive (case and contextual) based on the nature and method of data collection. It utilizes a mixed approach (quantitative and qualitative) due to because the fact that it is supposed to be deeply researched in a specific case. In this research, in a wide range, experiments are addressed to indicate the efficiency of the suggested conceptual model, and the suggested technique has been compared and evaluated with a set of algorithms. These tests have been performed on specific test samples.

In this sense, in the continuation of this section, the evaluation criteria and studied indicators have been introduced. In the second section, the settings of the parameters in the experiments are shown. In the third section, the compared algorithms are shown. In the research findings section, the results of the experiments are shown, and the discussion and conclusion section shows the review of the compared and evaluated algorithms.

### 4.1. Evaluation Criteria and Studied Indicators

A simple method for evaluating the quality of a set of solutions is with quality indicators (QIs). Overall, QIs are categorized into the following six groups:

1—QIs for convergence

2—QIs for spread

3—QIs for uniformity

4—cardinality index

5—QIs for spread and uniformity

6—QIs for the first four sections.

There are two groups of convergence in QIs:

1. Evaluating the Pareto dominance relationship between solutions or sets.
2. Evaluating the distance of a set of solutions from the beam view.

Generational distance (GD) determines the mean squared Euclidean distance of the solutions set for the nearest point on the ray. Spread quality is associated with the area coverage of a solution area. Pure diversity (PD) shows the variation of all solutions to the rest of the solutions in a set of solutions. The quality index for uniformity evaluates the uniform distribution of a set of solutions and measures the changes in the distance between solutions, i.e., spacing.

The cardinality index adds a diverse non-dominant solution to the set of solutions to progress the assessment. QIs for uniformity and spread are close to each other and can be used together to show the diversity of the set of solutions, and are categorized into two groups:

1. Indexes based on distance
2. Region division-based indicator

which partition a specific area into a large number of cells of the same size and then calculate the cell quantity that has solutions. Some of them consider the cells as grids that partition the space into a large number of hyper boxes, such as diversity metric.

The quality index covers convergence, diffusion, uniformity, and basic aspects for all aspects, and they are categorized into two groups:

A distance-based quality index that measures the distance of the beamformer to the set of considered solutions, such as inverted generational distance (IGD).

The volume-base index measures the size of the volume and is assigned to the set of considered solutions, such as hyper-volume (HV). Table 1 illustrates the connection between the assessment criteria and their grouping.

In this research, to evaluate the performance of the evolutionary MOA based on inverse modeling, seven samples with different numbers of IoT devices have been used: $n=\left[100,\;200,\;300,\;400,\;500,\;600,\;700\right]$.

### 4.2. Parameter Settings

It is considered that each IoT argument is randomly dispersed in a square meter of 1000 m by 1000 m, and the flying height of the drone is 200 m. Furthermore, $D_{i}$ is randomly distributed in the range of $\left[1,10^{3}\right]$ MB.

The initial value is determined randomly, and the values are M=5, $p_{i}=0.1\;w$ and $h_{0}=-30\;\mathrm{dB}$, $\sigma^{2}=-250\;\mathrm{dBm}$, and λ=9000. In each compared algorithm, the same parameter settings of the problems are used. A total of 20 independent executions for each comparison algorithm are performed on each of the test samples. The end disorder for all algorithms is a maximum of 95,000 fitness evaluations (FE) for all test samples. Wilcoxon rank sum test is equal to 10, and the size of the grouping model is equal to L=3, which is used to compare the obtained results at a significance level of 0.05 of the tested algorithms.

By taking the MOSHO as a reference algorithm in the Wilcoxon rank sum test, the label “+” means that the performance of the MOSHO algorithm is better than other comparative algorithms, the “−” label defines that the performance of the MOSHO algorithm is weaker than the other evolutionary algorithms, and the “≈” label means that they have almost the same result. In this study, the MOSHO algorithm is compared with some other works, including the non-dominated sorting genetic algorithm (NSGA) [29], the ant colony optimization (ACO) [29], and the multiple ant colony non-dominated sorting genetic algorithm II (MAC-NSGA II) [30]. The parameter values of the algorithms is given below:

- NSGA-II and MAC-NSGA II:

Pop-size: 85; max iteration: 200; crossover prob: 0.8; mutation prob: 0.2; mutation operator: 20; crossover operator: 20.

- ACO

Ant size: 85; max iteration: 200; evaporation rate: 0.1; No. deposited pheromone: 0.2; pheromone factor: one; heuristic factor: one.

### 4.3. Discussions

Here, the results of the experiments are shown and discussed. The statistical results are presented for each algorithm under consideration based on the function of the problem shown in Equation (27) and using the criteria of the expression evaluation indicators. They were evaluated on the PlatEMO and are shown in Table 2.

The results listed in Table 2 show the algorithms’ superiority according to the energy consumption of the IoT’ information collection system with the help of a drone. Furthermore, the MOSHO algorithm has the best performance (lowest energy consumption) on evaluation functions. As well as this, based on the Wilcoxon rank sum test used in this table, the signs ≈, −, and + compare the performance of each algorithm with respect to the MOSHO algorithm. Therefore, by examining the Table, it can be seen that the MOSHO algorithm performs better than the other evolutionary MOAs in seven evaluation functions. Finally, it is concluded that with rising in the number of IoT devices, the efficiency of the MOSHO algorithm is still better.

**Figure 3:**
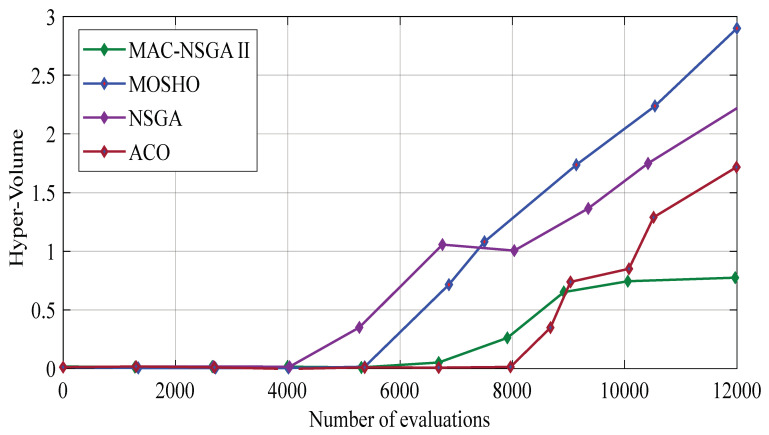
Hyper-volume results on the MOSHO and the associated studied techniques.

Figure 3 shows the graph of the hyper-volume outcomes on the MOSHO algorithm and the compared methods. It shows that the MOSHO method has a better performance in terms of convergence, diversity, and power with respect to the compared methods in the optimization of the problem. In fact, the super-volume criterion evaluates the degree of convergence, diversity, and the strength of the problem solutions by algorithms. Moreover, the MOSHO solution and comparative algorithms have been evaluated by the multi-volume criterion, and as shown in Figure 3, the MOSHO algorithm shown in blue has the best diversity, convergence, and power compared withother comparative algorithms.

## 5. Conclusions

In this study, a conceptual model for a bio-inspired MOSHO was used on the platform of data collection for a group of IoT devices around the earth. The performance of the MOSHO was evaluated and compared with other MOAs including NSGA, ACO, and MAC-NSGA II, and the results obtained are based on a comprehensive evaluation criterion that covers all of the necessary categories (convergence, diversity, and robustness). It has been shown that the MOSHO based on inverse modeling using Gaussian processing has increased the rate of convergence, diversity, and robustness of the problem compared with the evolutionary MOAs under investigation. Moreover, the MOSHO algorithm had the lowest energy consumption on evaluation functions. Due to the fact that the Pareto optimality of the solutions cannot be guaranteed, the fundamental drawback of using multi-objective optimization based on the MOSHO is its slower speed. Only the fact that none of the created solutions is superior to the others is known. In the future, to provide a better method for covering the MOSHO’s drawback, a new multi-objective optimizer with a simpler structure will be used.

## Figures and Tables

**Table 1:** The connection between assessment criteria and their classification.

Class	Inversed Generational Distance	Diversity Metric	Pure Diversity	Hyper- Volume	Spacing	Generational Distance
Convergence	*			*		*
Diversity	*	*	*	*		
Cardinality				*		
Uniformity		*			*	

**Table 2:** Energy consumption (J) using different algorithms.

N	MOSHO	NSGA	ACO	MAC-NSGA II
100	$1.38\times 10^{6}$	$1.42\times 10^{6}$	$1.66\times 10^{6}$	$1.45\times 10^{6}$
200	$2.57\times 10^{6}$	$2.61\times 10^{6}$	$2.85\times 10^{6}$	$2.67\times 10^{6}$
300	$3.74\times 10^{6}$	$3.86\times 10^{6}$	$4.21\times 10^{6}$	$3.72\times 10^{6}$
400	$5.16\times 10^{6}$	$5.30\times 10^{6}$	$5.55\times 10^{6}$	$5.36\times 10^{6}$
500	$6.14\times 10^{6}$	$6.23\times 10^{6}$	$7.50\times 10^{6}$	$6.31\times 10^{6}$
600	$7.50\times 10^{6}$	$7.78\times 10^{6}$	$8.38\times 10^{6}$	$7.95\times 10^{6}$
700	$8.04\times 10^{6}$	$8.16\times 10^{6}$	$8.90\times 10^{6}$	$8.53\times 10^{6}$
+		7	7	7
−		0	0	0
≈		0	0	0

## Data Availability

Not applicable.
